# Dose-Dependent Dual Effect of the Endozepine ODN on Neuronal Spiking Activity

**DOI:** 10.3390/brainsci15080885

**Published:** 2025-08-20

**Authors:** Mahmoud Hazime, Marion Gasselin, Michael Alasoadura, Juliette Leclerc, Benjamin Lefranc, Magali Basille-Dugay, Celine Duparc, David Vaudry, Jérôme Leprince, Julien Chuquet

**Affiliations:** 1 Univ Rouen Normandie, Inserm, Normandie Univ, NORDIC UMR 1239, F-76000 Rouen, Francemichael.alasoadura@jax.org (M.A.); magali.basille@univ-rouen.fr (M.B.-D.); celine.duparc@univ-rouen.fr (C.D.); david.vaudry@inserm.fr (D.V.); jerome.leprince@univ-rouen.fr (J.L.); 2 Univ Rouen Normandie, Normandie Univ, GRHVN UR3830, F-76000 Rouen, France

**Keywords:** endozepine, astrocytes, GABA, neuropeptides

## Abstract

Background/Objectives: Endozepines known as the endogenous ligands of benzodiazepine-binding sites, include the diazepam binding inhibitor (DBI) and its processing products, the triakontatetraneuropeptide (TTN) and the octadecaneuropeptide (ODN). Despite indisputable evidence of the binding of ODN on GABA_A_R-BZ-binding sites, their action on this receptor lacks compelling electrophysiological observations, with some studies reporting that ODN acts as a negative allosteric modulator (NAM) of GABA_A_R while others suggest the opposite (positive allosteric modulation, PAM effect). All these studies were carried out in vitro with various neuronal cell types. To further elucidate the role of ODN in neuronal excitability, we tested its effect in vivo in the cerebral cortex of the anesthetized mouse. Methods: Spontaneous neuronal spikes were recorded by means of an extracellular pipette, in the vicinity of which ODN was micro-infused, either at a high dose (10^−5^ M) or low dose (10^−11^ M). Results: ODN at a high dose induced a significant increase in neuronal spiking. This effect could be antagonized by the GABA_A_R-BZ-binding site blocker flumazenil. In sharp contrast, at low concentrations, ODN reduced neuronal spiking with a magnitude similar to GABA itself. Interestingly, this decrease in neuronal activity by low dose of ODN was not flumazenil-dependent, suggesting that this effect is mediated by another receptor. Finally, we show that astrocytes in culture, known to be stimulated by picomolar doses of ODN via a GPCR, increased their export of GABA when stimulated by low dose of ODN. Conclusion: Our results confirm the versatility of ODN in the control of GABA transmission, but suggest that its PAM-like effect is, at least in part, mediated via an astrocytic non-GABA_A_R ODN receptor release of GABA.

## 1. Introduction

Pioneering research on the effect of benzodiazepines (BZs) on GABA_A_ receptor (GABA_A_R) function has led to the discovery of endogenous BZ receptor ligands designated by the generic term «endozepines». Endozepines are a family of peptides including the diazepam binding inhibitor (DBI, also known as Acetyl Co-A Binding Protein, ACBP) and its processing products, the octadecaneuropeptide (ODN), a small peptide of 18 amino-acids (DBI 33-50), and the triakontetraneuropeptide (TTN, DBI 17-50) [1]. Endozepines are produced by astrocytes [2,3,4] in most central nervous system structures, including the cerebral cortex, the hippocampus, the cerebellum, and the spinal cord [1,5]. Despite compelling evidence of the binding of DBI and ODN on GABA_A_R-BZ-binding sites, with K*i* reported between 1 and 5 μM [6,7,8,9], there is no direct information on the precise subunit composition of endozepine-sensitive GABA_A_Rs [1,10,11,12,13]. The existence of another type of receptor, which likely belongs to the GPCR family, has been repeatedly proposed to explain the flumazenil-insensitive biological action of ODN [14,15,16,17,18].

The function of endozepine peptides on GABA_A_R has been inferred from numerous neurophysiological (i.e., electroencephalography) or behavioral experiments in which (i) these peptides mimic the effects of other GABA_A_R allosteric modulators [6,19,20,21] and (ii) these effects can be blocked by the BZ antagonist flumazenil. However, the direct electrophysiological effect of exogenously applied endozepine peptides on GABA-mediated inhibitory currents in neurons has rarely been investigated. The few available experiments on the effects of some endozepines (ODN, essentially) on neuronal activity have been carried out in cultured cells or in brain slices using various types of neurons (e.g., embryonic spinal motoneurons, neural progenitors). From these in vitro preparations, it was reported that endozepines act as negative allosteric modulators (NAMs) of the GABA_A_-R, i.e., reducing the GABA hyperpolarizing current [6,19,20,21]. This effect was nearly abolished in mutant mice carrying a phenylalanine (F) to isoleucine (I) substitution at position 77 in the N-terminal domain of the γ2 subunit, rendering the GABA_A_R insensitive to BZs (γ2-F77I mice) [6,22]. Of note, all these studies used fairly high concentrations of ODN, in the micromolar range. Following another approach presented in a series of recent publications, Christian and colleagues reported a much more nuanced view of the role of endozepines in the modulation of GABA neurotransmission [23,24]. Using *DBI* KO (DBI^−^/^−^) mice, the authors observed that miniature IPSC frequency and amplitude in CA1 pyramidal cells are increased compared to wild-type mice, in agreement with the NAM effect of DBI and ODN described above [24]. However, the opposite observation was made in granule cells of the dentate gyrus, where the loss of DBI decreased miniature IPSC amplitude and increased their decay time, suggesting that in this subregion of the hippocampus, DBI or its processing products act as positive allosteric modulators (PAMs) of GABA_A_R [24]. The regional specificity of the action of DBI (NAM vs. PAM) was also suggested by earlier studies on the TRN [23]. Another discrepancy between the expected NAM action of ODN and its effect on synaptic plasticity is that neurons overexpressing DBI show a surprising decrease in LTP [25]. To further elucidate the role of endozepine in neuronal GABA inhibition, we tested the effect of ODN in vivo in the cortex of the anesthetized mouse. We confirm previous in vitro studies showing that at micromolar concentrations, ODN acts as a NAM of GABA_A_-R, increasing the frequency of neuronal firing in a flumazenil-dependent manner. In sharp contrast, at low concentration (10^−11^ M), ODN reduced neuronal firing, an effect insensitive to flumazenil. As ODN is known to activate astrocytes at low concentrations in a flumazenil-insensitive manner, we further explored whether ODN is involved in the control of GABA release/uptake. In agreement with in vivo observations, we found that ODN at low but not high concentrations triggers GABA release by astrocytes.

## 2. Material and Methods

### 2.1. Study Approval

Experiments, approved by the Ethics Committee for Animal Research of Normandy (Institution No. 76-451-04, project 03314.01, approved 15 March 2017) were conducted by authorized investigators in accordance with the recommendations of the European Communities 86/609/EEC.

### 2.2. Animals

For these studies, 8–12-week-old (20–25 g) male C57BL/6J mice were purchased from Janvier Laboratories. Animals were housed with free access to standard laboratory diet and tap water, under controlled temperature (22 ± 1 °C) and lighting (light from 7:00 a.m. to 7:00 p.m.). Newborn (24–48 h) C57BL/6 mice were used to prepare secondary cultures of mouse cortical astrocytes for in vitro experiments. All procedures and reporting were undertaken in accordance with the ARRIVE (Animal Research: Reporting In Vivo Experiments) guidelines.

### 2.3. In Vivo Electrophysiology

Under isoflurane anesthesia (2–2.5%), two small holes were drilled over the whisker barrel cortex with the dura matter intact and two glass micropipettes were inserted (one for the recording and one for the micro-injection). After the surgical procedure, isoflurane was reduced to 1.1 ± 0.1% for a resting period of 45–60 min, followed by the recording period. All in vivo recordings were performed in a Faraday chamber and used an amplifier PowerLab 8/35 (AD-Instrument, Sydney, Australia). Raw data were acquired with Labchart software (AD Instrument).

### 2.4. Extracellular Unit (EU) Recording

An ACSF-filled glass micropipette/AgCl/Ag electrode, 3–6 µm tip diameter opening, was positioned in cortical layer 4 of the whisker barrel cortex. Reference and ground electrodes (AgCl/Ag coated wire) were inserted into the cerebellum. For ODN microinjection, a second glass micropipette (10 µm tip diameter opening) was placed 30–50 µm away from the tip of the recording pipette. The signal was bandpass-filtered at 200–2000 Hz and digitized at 20 kHz for EU. Signals were recorded for 10 min before the intracortical microinjection of 0.5 µL of ODN at 10^−5^ or 10^−11^ M and compared to a 10 min period beginning 3 min after the end of the microinjection (3 min). Spike detection and sorting were then performed semi-automatically, using Klusta software suite [26], freely available on the internet (http://klusta-team.github.io, accessed on23 April 2019).

### 2.5. Peptide Synthesis

Mouse/rat ODN (H-Gln-Ala-Thr-Val-Gly-Asp-Val-Asn-Thr-Asp-Arg-Pro-Gly-Leu-Leu-Asp-Leu-Lys-OH) was synthesized as previously described [15].

### 2.6. Astrocytes Culture

Secondary cultures of mouse cortical astrocytes were prepared as previously described [27] with minor modifications. Briefly, cerebral hemispheres from newborn C57BL/6 mice were collected in DMEM/F12 (2:1; *v*/*v*) culture medium supplemented with 2 mM l-glutamine, 1‰ insulin, 5 mM HEPES, and 1% of the antibiotic–antimycotic solution. The tissues were dissociated mechanically with a syringe equipped with a 1 mm gauge needle and filtered through a 100 µm sieve (Falcon). Dissociated cells were resuspended in culture medium supplemented with 10% FBS, plated in 75 cm^2^ flasks (Greiner Bio-one GmbH, Frickenhausen, Germany), and incubated at 37 °C in a 5% CO_2_/95% O_2_ atmosphere. When cultures were confluent, astrocytes were isolated by shaking overnight the flasks on an orbital agitator. Adhesive cells were detached by trypsinization and seeded for 5 min to discard contaminating microglial cells. Then, the non-adhering astrocytes were harvested and plated in 24-well plates with an average density of 80,000 cells/mL. All experiments were performed on 48-to-72-h-old secondary cultures (80% confluence). In these conditions, more than 97% of cells were labeled with antibodies against glial fibrillary acidic protein [28].

### 2.7. [^3^H]GABA Uptake Assay

The measurement of [^3^H] GABA uptake by astrocytes was based on the Fattorini et al. protocol [29]. The plates (12 wells) were sequentially incubated in a control medium (0.6% DMSO), a medium containing a selective GAT-1 inhibitor, i.e., tiagabine (50 μM, Sigma Aldrich, St. Louis, MO, USA), or a selective GAT-3 inhibitor, i.e., SNAP-5114 (30 μM, Sigma Aldrich), after which ODN (0, 10–6, 10–9, and 10–12 M) was added for 15 min at 37 °C. In the subsequent step, astrocytes were incubated with a mixture of 30 μM of GABA and [^3^H] GABA (40 μCi, specific activity = 25 Ci/mmol, PerkinElmer, Waltham, MA, USA, 15 min, 37 °C). The supernatant was then removed, the reaction halted by washing with PBS (200 μL, 0.1 M), and the cells lysed in a lysis solution (200 μL, 2.5 mL Tris, 5 mL SDS 10%, and 42.5 mL distilled water) and mixed with a scintillator liquid (Ultima Gold, Revvity Health Sciences Inc., Burlington, MA, USA, 3 mL). The final solution obtained was passed to the liquid scintillation counter (Tricarb, PerkinElmer, Waltham, MA, USA 2800 TR) in order to quantify the radioactivity in the cells.

### 2.8. GABA Release Assay

To measure the release of GABA from cultured astrocytes, we used the Mouse GABA ELISA Kit (Abbexa, Ltd., Cambridge, UK) according to the manufacturer’s protocol. Astrocyte cultures were treated with 100 µL of either vehicle (control) or ODN at different concentrations (10^−6^ M or 10^−12^ M). At defined time points after treatment (0, 5, 10, 20, and 30 min), the extracellular medium was collected and stored at −80 °C. GABA concentration in the supernatant was then quantified using the ELISA kit. Absorbance was measured at 450 nm using a microplate reader (Infinite 200, TECAN, Männedorf, Switzerland). Each condition was performed in triplicate wells per culture, across three independent cultures.

### 2.9. Summary of Drugs and Chemicals

For in vivo micro-infusion experiments, ODN was applied locally at 10^−5^ M or 10^−11^ M in 0.5 µL of aCSF. In astrocyte assays, ODN was used at concentrations of 10^−6^ M, 10^−9^ M, or 10^−12^ M for GABA uptake experiments, and at 10^−6^ M and 10^−12^ M for GABA release assays. Flumazenil was purchased from Sigma-Aldrich and dissolved in sterile HEPES buffer supplemented with KCl (2.5 mmol/L) and NaCl (145 mmol/L) pH 7.4 and DMSO (dilution 1:4), or in culture medium at 0,6% for in vitro assays. GABA (Sigma Aldrich) was applied at 10 mM in vivo as a positive control (0.5 µL injection) and at 30 µM in uptake assays, where it was co-incubated with 40 µCi [^3^H]GABA (PerkinElmer, NET-091). The uptake assays also included pharmacological inhibitors, tiagabine hydrochloride (T-9754) at 50 µM and SNAP-5114 (S-9068) at 30 µM, both prepared as stock solutions in culture medium.

### 2.10. Statistics

Data are presented as mean ± SEM. Normalized spiking rates obtained by cumulating the number of spikes per 10-s bin from the raster plot were then all normalized relative to the average number of spikes during the 10 min PRE period, which was set to 100. All statistics were analyzed using Prism software (Graphpad, version 9.0.0). Normal distribution of the datasets was tested by a Kolmogorov–Smirnov test. A paired Student’s *t*-test was used for pairwise means comparisons. A Wilcoxon signed rank test was used for pairwise means comparisons of data that did not follow normal distribution. An adjusted *p* value was calculated using the false discovery rate (Benjamini–Hochberg) method when multiple comparison tests were performed.

## 3. Results

**Figure 1:**
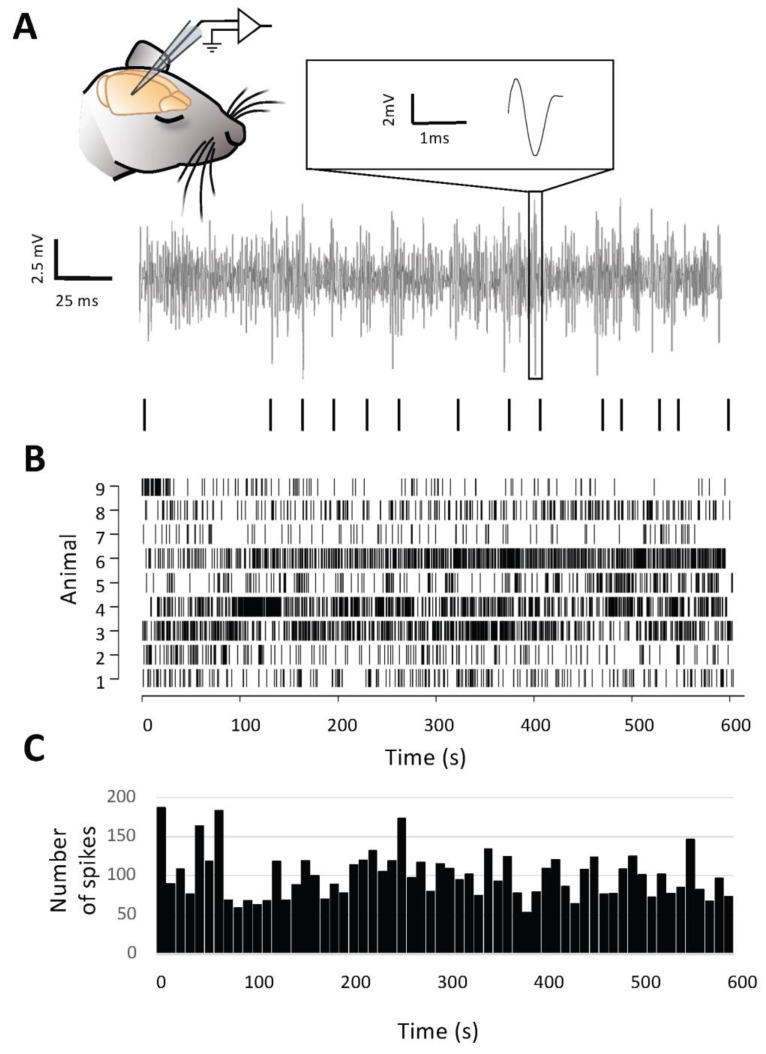
Extracellular in vivo recording of neuronal activity. (**A**). In vivo extracellular recording of cortical neuronal spiking. Representative extracellular unit recording trace of spontaneous neuronal spiking activity. The occurrence of spikes is depicted by short black bars underneath the trace. (**B**). Raster plot of neuronal spiking recorded in 9 mice over 10 min. (**C**). Spiking rate obtained by cumulating the number of spikes per bin of 10 s from the raster plot.

**Figure 2:**
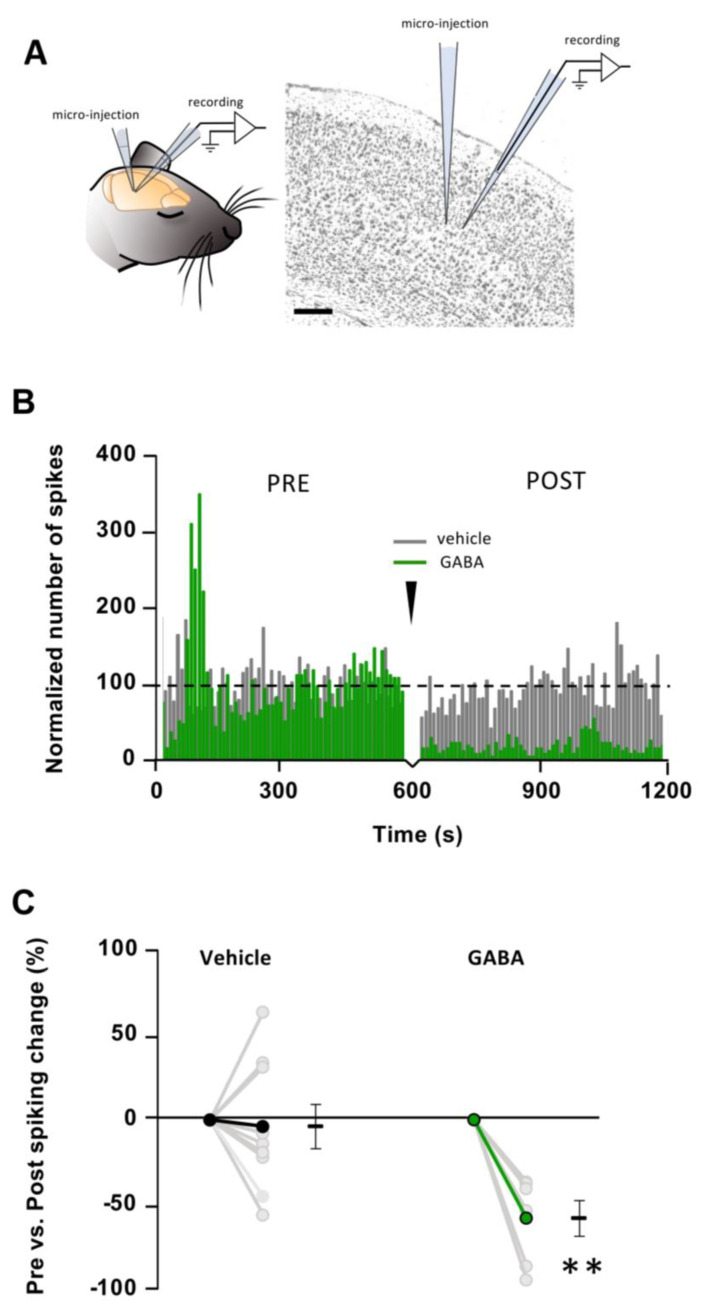
Controls: effects of vehicle and GABA on cortical spiking rate. (**A**). Experimental arrangement showing the double implantation of pipettes for micro-injection of ODN in the vicinity (~50 μm) of the recording pipette (layer 4; scale bar: 100 μm). (**B**). Effect of the micro-injection of vehicle (gray line) or GABA (green line) on neuronal spiking rate (Black arrowhead marks the onset of the microinjection). Overall, spiking rate was stable, and not influenced by the administration procedure. Neurons answered to GABA (200 μM) by a strong reduction in their activity. (**C**). Spiking rate change per animal and average. Vehicle: PRE vs. POST, *p* > 0.05; *n* = 9 mice. GABA: PRE vs. POST, *p* < 0.01 (**); *n* = 6 mice.

In order to probe the effect of exogenous ODN on cortical neuron activity in vivo, we designed a protocol in which ODN was administrated into the cortex with limited mechanical perturbation likely to create a bias. To validate this original approach, three controls were necessary. The first was being able to obtain a stable firing rate serving as a baseline (Figure 1A–C). After a stabilization period of 45 min under 1.1 ± 0.1% of isoflurane with continuous monitoring of the LFP activity, we found that 10 min was a necessary albeit sufficient duration to obtain a reliable baseline spiking rate (Figure 1C). The second prerequisite was to establish a microinjection method that would not induce any perturbation of the locally recorded activity. After a first period of 10 min, defined as “PRE”, 0.5 µL of aCSF (vehicle) was slowly (2.8 nL/s) pressure-ejected through a glass micropipette, the tip of which (3 to 6 µM in diameter) was positioned 30–50 µm away from the recording micropipette (Figure 2A). A second period of 10 min of recording (defined as “POST”) started at the end of the 3 min infusion period. Using this arrangement and these injection parameters, aCSF administration did not change the spiking rate between PRE and POST periods (4 ± 13% change from PRE-aCSF baseline; *n* = 9 mice; *p* > 0.05) (Figure 2B,C). The third prerequisite was to set a positive control to assess the sensitivity of our in vivo assay. To this end, we microinjected 10 mM of GABA while recording neuronal firing. As expected, GABA depressed neuronal activity (−58 ± 10% change from PRE-GABA baseline; *n* =6 mice; *p* < 0.01) (Figure 2B,C).

**Figure 3:**
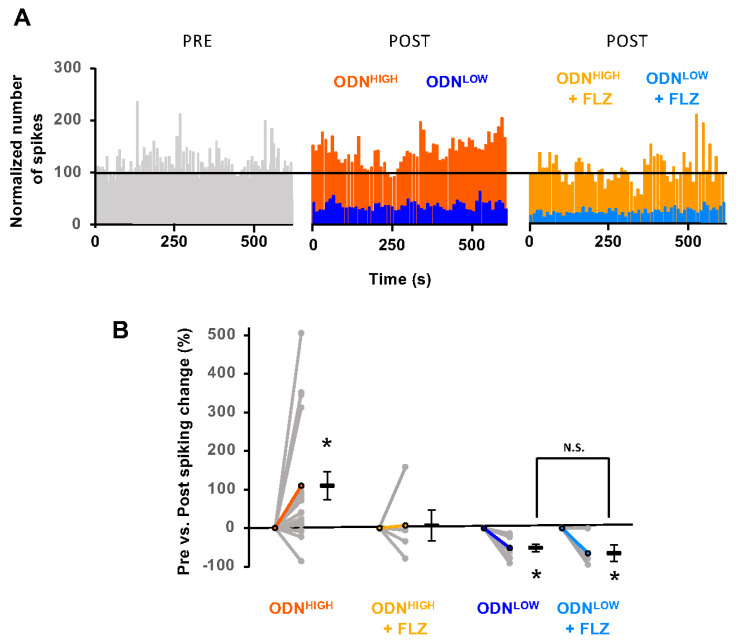
Bi-directional effect of the gliopeptide ODN on neuronal spiking. (**A**). Effects of the micro-injection of ODN at high concentration (10^−5^ M; ODN^High^) without and with flumazenil (FLZ) or at low concentration (10^−11^ M; ODN^Low^) on neuronal spiking rate. (**B**). Spiking rate change per animal and average for each condition. ODN^High^: PRE vs. POST *p* < 0.05; *n* = 19 mice. This effect was abolished with flumazenil (*n* = 5 mice; PRE vs. POST, *p* > 0.05). ODN^Low^: PRE vs. POST, *p* < 0.05; *n* = 8 mice. This effect was insensitive to flumazenil (PRE vs. POST, *p* < 0.05; *n* = 5 mice). Stars (*) indicate a significant difference in spiking rate compared to the “PRE” condition (10 min baseline). N.S.: not significant.

The neuropeptide ODN at high concentration (10^−5^ M; ODN^High^) induced an increase in spiking rate, with high inter-experiment variability (110 ± 36% change from PRE-ODN^High^ baseline; *n* = 19 mice; *p* < 0.05; Figure 3A,B). This effect was abolished by the GABA_A_R-BZ site antagonist flumazenil (FLZ) (7 ± 40% change from PRE-ODN^High^/FLZ baseline; *n* = 5 mice; *p* > 0.05). At low concentration (10^−11^ M; ODN^Low^), ODN significantly decreased the spiking rate (−51 ± 9% change from PRE-ODN^Low^ baseline; *n* = 8 mice; *p* < 0.05). This effect was insensitive to flumazenil (−64 ± 21% change from PRE-ODN^Low^/FLZ baseline; *n* = 5 mice; *p* < 0.05; *p* > 0.05 relative to POST-ODN^Low^; Figure 3A,B).

ODN is a gliopeptide exclusively synthetized by astrocytes [1]. At low concentrations (10^−9^ to 10^−15^ M), ODN is known to stimulate intracellular calcium release of astrocytes in culture [30], and increase their spontaneous calcium surge frequency both in vitro [28] and in vivo [31]. Furthermore, astrocytes are involved in GABA uptake and release to such an extent that this can influence GABAergic transmission [32]. To test the hypothesis that the ODN-induced neuronal activity change is mediated by astrocytes, we tested the effect of ODN on GABA uptake and release by mouse cortical astrocytes in pure culture.

**Figure 4:**
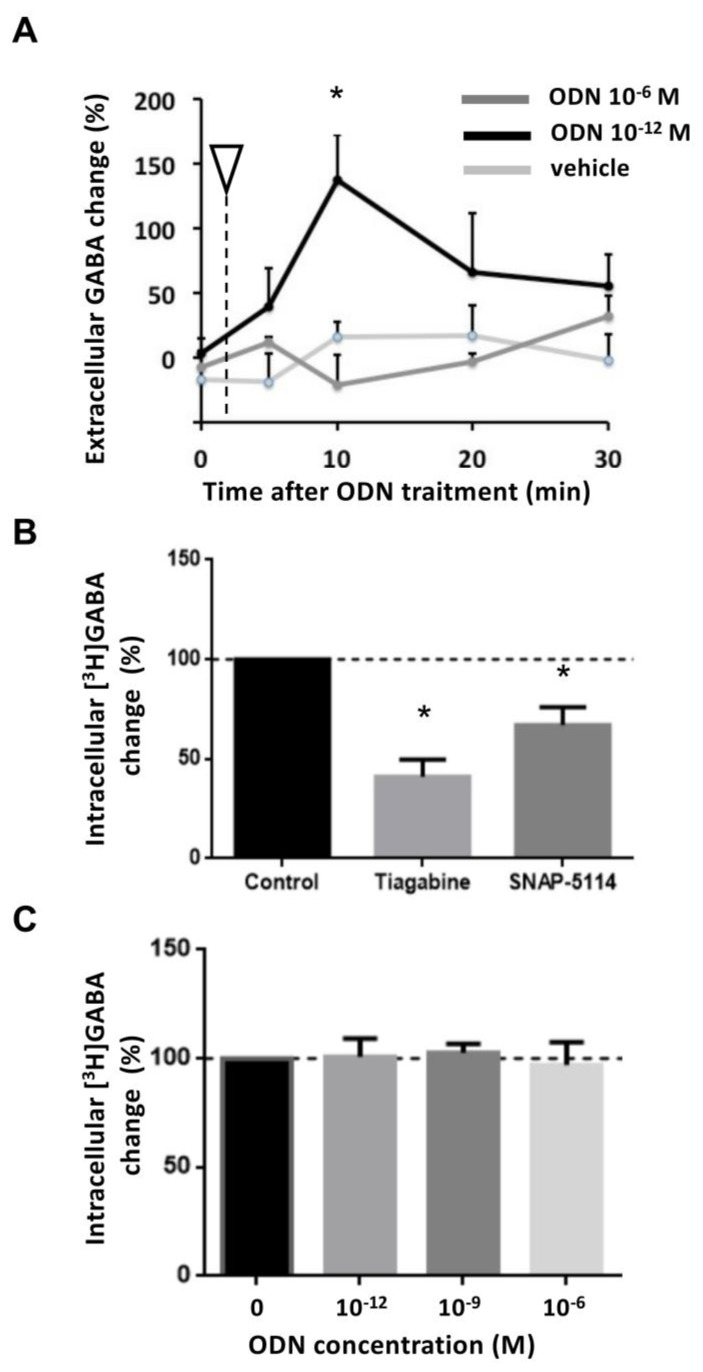
Effect of ODN on GABA release and uptake by astrocytes in culture. (**A**). Effect of ODN on GABA release by astrocytes. Vehicle (light gray line) and ODN^High^ (10^−6^ M, dark gray line) had no effect on the concentration of GABA in the astrocyte culture medium (*p* > 0.05 at any time point for both vehicle and ODN^High^, *n* = 3 cultures, with three wells per condition). ODN^Low^ (10–12 M, black line) induced a sharp rise in the extracellular concentration of GABA, which reached a peak 10 min after the administration (*p* < 0.05 (*); *n* = 3 cultures, with three wells per condition). (**B**). Positive control for [^3^H]GABA uptake by astrocytes. Tiagabine and Snap-5114, two inhibitors of the GABA transporters GAT-1 and GAT-3, respectively, significantly decreased the uptake of [^3^H]GABA (*p* < 0.05 (*) for both inhibitors relative to control; *n* = 4 independent experiments). (**C**). ODN failed to modify the transport of [^3^H]GABA regardless of the concentration used (*p* > 0.05 for the concentration of ODN, *n* = 4, *n* = 6, *n* = 3, independent experiments, respectively).

The influence of high (10^−6^ M) and low (10^−12^ M) [ODN] on the release of GABA by astrocytes was first studied using an ELISA assay. The infusion of the vehicle medium (100 µL) did not elicit any significant changes in extracellular [GABA] at any time point (*n* = 3 cultures with three wells/culture for each condition; *p* > 0.05; Figure 4A). Similarly, ODN at high concentration (10^−6^ M) did not change the extracellular amount of GABA (*n* = 3; *p* > 0.05; Figure 4A). In contrast, low [ODN] (10^−12^ M) rapidly induced an elevation in extracellular [GABA], reaching significance at 10 min post-infusion (137 ± 35.4% relative to pre-ODN value; *p* < 0.05; Figure 4A).

Finally, to address the possibility that ODN modifies astrocytic GABA uptake, we used a radio-assay to quantify the intracellular accumulation of [^3^H]GABA. The uptake of [^3^H]GABA in the presence of the selective GAT-1 inhibitor tiagabine (50 μM) or the selective GAT-3 inhibitor SNAP 5114 (30 μM) was significantly reduced by 59 ± 9% and 33 ± 9%, respectively (*n* = 6 and *n* = 3, respectively; *p* < 0.05; Figure 4B) relative to the total uptake over 30 min in the control condition. However, ODN failed to modify the transport of [^3^H]GABA into astrocytes for the three concentrations of ODN tested, 10^−6^, 10^−9^, and 10^−12^ M, (*n* = 4; *n* = 6; *n* = 3, respectively; *p* > 0.05; Figure 4C).

## 4. Discussion

The general purpose of this study was to test the effect of the endozepine ODN on neuronal activity in vivo for the first time. Our hypothesis was that the apparent discrepancy reported in the literature about the PAM vs. NAM activity of endozepine for the GABA_A_R may lie in the concentrations at which these peptides operate. In fact, most experiments consistent with a GABA_A_R-NAM effect of ODN have used a µM-to-mM concentration of peptide [21,33], while many in vitro and in vivo studies bring evidence that the neuropeptide ODN works at very low concentration, in the fM-to-pM range [16,17,34].

This study relies on an original in vivo approach aimed at testing the direct and local electrophysiological effect of ODN at different concentrations. A double implantation set-up, leaving the recording and injecting pipette tips 30–50 µm apart, was achievable in the cortical gray matter, known to express the endozepine system and most GABA_A_ receptor subtypes.

The first finding of the present work is that ODN increases or decreases neuronal activity, depending on the concentration used. In agreement with all in vitro electrophysiological studies testing the effect of exogenously applied ODN in the micromolar range (no other dosage was actually tried), we found an elevation in neuronal activity, blocked by flumazenil, consistent with a GABA_A_R NAM effect. Surprisingly, we found the exact opposite when ODN was micro-infused at 10 pM into the cortex, a reduction in the neuronal spiking rate, consistent with the GABA_A_R PAM effect suggested in several recent publications [23]. It is tempting to believe that the lowest ODN concentration may be physiologically relevant, while the µM-to-mM range is never reached in real in vivo conditions. However, between brain cells, interstitial volume is extremely narrow [35] allowing the concentration of neurotransmitters or other exocytosed molecules to locally reach the mM range. For instance, Overstreet and Westbrook [36] estimated that GABA reaches a synaptic cleft concentration of 3–5 mM. It is therefore conceivable that ODN, in the close vicinity of its site of release from an astrocytic process, modulates the GABA_A_-R with a NAM effect.

Of note, since this observation was conducted in male mice, future studies will aim to assess whether the observed effects of ODN are conserved or modulated across sexes.

The second important finding of this study is the insensitivity of low concentrations of ODN for flumazenil. The decrease in neuronal activity that we observed is unlikely to be mediated by an interaction of ODN with the GABA_A_R. Indeed, ODN is known to operate at low concentrations via a GPCR, the identity of which remains to be elucidated [1,18,37]. Interestingly, ODN-induced calcium elevation in astrocytes is insensitive to flumazenil and has its optimal effect in the pM range. Moreover, at 10^−6^ M and above, ODN is no more efficient at inducing intracellular calcium transient in astrocytes [28]. Altogether, these similitudes led us to hypothesize that the observed GABA_A_R-PAM-like effect could actually be the result of an inhibition mediated by astrocytes. There is now a large body of evidence that supports the involvement of astrocytes in the modulation of the GABAergic transmission [32]. In particular, the control of extracellular GABA concentration through the regulation of GABA transport to or from astrocytes can influence the local neuronal excitability [38,39,40]. To address the relevance of our hypothesis, we used an in vitro approach with cortical astrocytes in culture. We found that ODN has no effect on the uptake of GABA. Conversely, ODN stimulated the release of GABA, at low but not high concentrations. This result supports the view that ODN is not a GABA_A_R PAM, but can nonetheless act to amplify GABA transmission, likely via its GPCR, expressed by astrocytes. Following this idea, ODN at a low concentration would enhance astrocyte-mediated tonic inhibition [1,41]. Further experiments are necessary to validate this hypothesis. For example, it would be interesting to verify in pure neuronal cultures whether low concentrations of ODN contribute to GABA release. This seems unlikely, as the literature lacks any evidence of a neuronal response to low ODN concentrations in the absence of astrocytes [1].

The function of endozepines remains elusive [12]. However, an array of arguments lets us hypothesize that endozepines are instrumental for gliotransmission at the inhibitory synapse. First, DBI is not only specifically produced by astrocytes but is also one of the most transcripted genes in astrocytes [42]. Second, potassium depolarization stimulates ODN release from astrocytes [30]. Third, both ODN and GABA provoke an elevation in cytosolic calcium in astrocytes [28,32]. Fourth, extrasynaptic GABA_A_-R is sensitive to BZ and ideally located to bind ODN released by local astrocytic processes. It is therefore tempting to propose a mechanism in which astrocytes sense synaptically released GABA [39,40] and respond by a release of ODN to modulate positively or negatively tonic inhibition. The present observations make the peptide ODN an appealing candidate as a gliomodulator of the inhibitory synapse.

## Data Availability

The raw data supporting the conclusions of this article will be made available by the authors on request.
